# Intron-Mediated Enhancement: A Tool for Heterologous Gene Expression in Plants?

**DOI:** 10.3389/fpls.2016.01977

**Published:** 2017-01-06

**Authors:** Miriam Laxa

**Affiliations:** Institute of Botany, Leibniz University HannoverHannover, Germany

**Keywords:** intron-mediated enhancement, tissue specificity, plants, gene expression, transcription, crop improvement

## Abstract

Many plant promoters were characterized and used for transgene expression in plants. Even though these promoters drive high levels of transgene expression in plants, the expression patterns are rarely constitutive but restricted to some tissues and developmental stages. In terms of crop improvement not only the enhancement of expression *per se* but, in particular, tissue-specific and spatial expression of genes plays an important role. Introns were used to boost expression in transgenic plants in the field of crop improvement for a long time. However, the mechanism behind this so called intron-mediated enhancement (IME) is still largely unknown. This review highlights the complexity of IME on the levels of its regulation and modes of action and gives an overview on IME methodology, examples in fundamental research and models of proposed mechanisms. In addition, the application of IME in heterologous gene expression is discussed.

## Introduction

**FIGURE 1:**
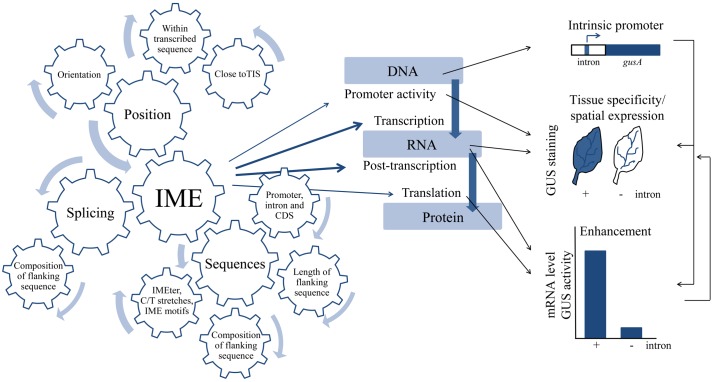
**Complexity of intron-mediated enhancement (IME) on both the level of regulation and the modes of action.** IME depends on positional requirements, sequences and elements within the intron and, in the case of monocots, on splicing. IME affects all levels of gene expression. IME can be measured indicate (intrinsic promote activity and enhancement) and visualized in different tissues (tissue specificity/spatial expression). Bent arrows in the gear-wheel indicate that all parameters influencing IME mainly have an additive affect rather than counter acting each other. Thickness of the arrows indicates the importance of the parameter for IME. Blue straight arrows indicate which level of gene expression is affected at a certain frequency (thickness correlates with the number of publications). Black straight arrows indicate modes of action of IME in dependence of both the level gene expression targeted and internal levels of relationship.

For a long time, gene regulation has mainly been attributed to *cis*-elements in the promoter regions of genes. However, since their discovery in 1977 (Sambrook, 1977), introns became an important player in gene regulation. Introns drive evolution by exon shuﬄing (Long et al., 1995) and enable translation of multiple proteins from a single gene by alternative splicing (Maniatis and Tasic, 2002). Additionally, introns initiate and enhance gene expression by a mechanism called intron-mediated enhancement (IME) not only in plants but also in mammals, insects, nematodes, and yeast (Callis et al., 1987; Okkema et al., 1993; Furger et al., 2002; Moabbi et al., 2012; Jiang et al., 2015). Even though IME was already shown in plants in 1987 (Callis et al., 1987) the mechanism of IME is largely unknown. This is due to the fact that IME is a complex phenomenon (**Figure 1**). Common to all introns that are involved in IME is that the introns must be located in a correct orientation (Vasil et al., 1989; McElroy et al., 1990; Maas et al., 1991; Rethmeier et al., 1997; Mun et al., 2002; Curi et al., 2005) within the transcribed sequence (Callis et al., 1987; Mascarenhas et al., 1990; Clancy et al., 1994) and close to the transcription initiation start (TIS; Rethmeier et al., 1997; Rose, 2004; Parra et al., 2011). IME is influenced by sequence elements. However, IME cannot be assigned to one specific sequence element and is more likely a result of a combination of multiple factors. Predominantly, a role for C/T-stretches was proposed to facilitate IME (Huang et al., 1997; Rose and Beliakoff, 2000; Clancy and Hannah, 2002; Mun et al., 2002; Jeong et al., 2007). Additionally, IME was linked to specific sequence motifs (TTNGATYTG, Rose et al., 2008; CGATT, Parra et al., 2011) that were found in a bioinformatic approach that was based on the observation that the nucleotide composition of introns located close to TIS is different to those located further downstream in the gene body (IMEter, Rose et al., 2008). IME also depends on the length and composition of sequences directly flanking the introns as well as on downstream coding sequences (CDS; Luehrsen and Walbot, 1991; Maas et al., 1991; Sinibaldi and Mettler, 1992; Clancy et al., 1994; Rethmeier et al., 1997). In addition, flanking sequences influence the splicing process of introns. IME in monocots necessitates splicing while it is of minor importance in dicots (Mascarenhas et al., 1990; Sinibaldi and Mettler, 1992; Jeon et al., 2000; Rose and Beliakoff, 2000; Clancy and Hannah, 2002; Rose, 2002; Morello et al., 2006; Akua et al., 2010).

As observed on the regulatory level, IME is as complex on the level of its action (**Figure 1**). IME affects all levels of gene expression, but the strongest effect of IME was shown on the levels of post-transcription and translation (Rose and Last, 1997; Samadder et al., 2008). However, a detectable effect on the translation level relative to the post-transcriptional level was only shown for a few introns (Mascarenhas et al., 1990; Lu et al., 2008; Samadder et al., 2008). IME rarely targets the promoter level (Salgueiro et al., 2000; Kim et al., 2006; Liao et al., 2013; Xiao et al., 2014) but should not be overseen because gene expression in *Caenorhabditis elegans* entirely depends on introns (Okkema et al., 1993). The last level of complexity is based on the observation that even though different levels are targeted by IME the effect can be very different. For example, an intron targeting the level of either post-transcription or translation can simply enhance expression of a gene on both levels, while an intron targeting the RNA level can impact either tissue specificity or the level of gene expression. This is very important because some introns not enhance expression but restrict expression to specific tissues (Liao et al., 2013). However, to date it is not clear whether changes in both tissue specificity and spatial expression of genes by IME can be attributed to the DNA or RNA level.

Heterologous gene expression in plants plays a role in optimizing yields and improving resistance to various biotic and abiotic pathogens. Alongside with bacterial and mammalian cells, plants can be used as expression systems for (i) therapeutic proteins, (ii) proteins used as reagents for research, and (iii) proteins that are suitable for industrial application (Desai et al., 2010). However, although tools and techniques of plant biotechnology are established, implementation beyond research is still rare. In their review, Desai et al. (2010) summarized advantages of plant expression systems. The expression of proteins in transgenic plants is advantageous because (i) production costs are lower, (ii) the post-translational modifications between plants and human are quite similar, (iii) expression can be easily scaled-up, (iv) storage costs are lower (for example when the protein is expressed in seeds), and (v) the risk of the spread of foreign proteins is lowered if the transgene is not expressed in pollen. However, the major limit of gene expression in transgenic plants is the low yield of final protein (Desai et al., 2010).

## Methodology of Studying IME

**FIGURE 2:**
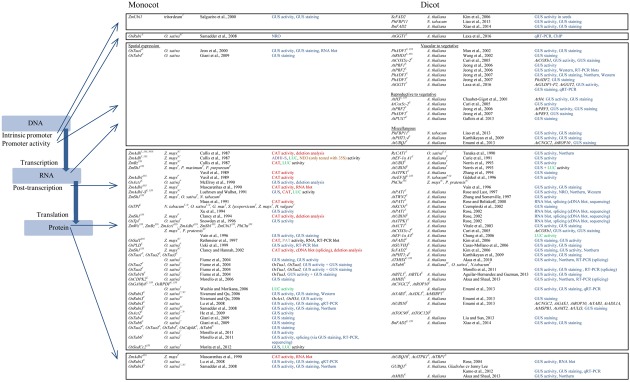
**Introns mediate the enhancement of gene expression in both monocots and dicots.** Arrows indicate the modes of action that either was reported for a specific intron or can be proposed as a mechanistic level for a group of introns. The list summarizes information on the given publications in the following way: (i) intron in combination with specific promoters (E – endogenous promoter studied, F – foreign promoter, 35S – CaMV 35S promoter, NOS – nopaline synthase promoter), (ii) plant species in which IME was tested in and the type of transformation (not specifically signed – stable transformation, P – protoplast, C – callus, SC – suspension cells, T – transient, for example in leaves, St – stable transformation, specifically indicated when multiple types of transformation were used in the study), (iii) reference, and (iv) methods and reporter genes used to test IME of the intron-promoter combination given. The different reporter genes are highlighted with a color code (blue – *gusA* encoding β-glucuronidase, red – *cat* encoding chloramphenicol acetyltransferase, green – *luc* encoding firefly luciferase, grey – *bar* encoding phosphinothricin acetyltransferase, brown – *neo* encoding neomycin phosphotransferase II, and purple – *Adh1-S* encoding ADH1-S). In case a foreign promoter was tested the gene name is given.

The finding that IME is important for gene expression coincided with the ability to use recombinant DNA technologies with regard to plant transformation (Malik, 1981). In the early 1980s, Barton et al. (1983) reported the regeneration of intact tobacco plants that were successfully genetically engineered by integrating a T-DNA into the plants’ genome. Application was further boosted by the establishment of binary *Agrobacterium* vectors (Bevan, 1984). In the late 1980s, reporter genes were developed to demonstrate gene expression in transient and stable transformation systems. Among those reporter genes used to study IME (**Figure 2**), the gene *nptII* encoding neomycin phosphotransferase II (NEO) was established first (Brzezinska and Davies, 1973), followed by *luc* encoding firefly luciferase (LUC) and *cat* encoding chloramphenicol acetyltransferase (CAT; Fromm et al., 1985; Ow et al., 1986). Soon after, *bar* encoding phosphinothricin acetyltransferase (PAT) and *gusA* encoding β-glucuronidase (GUS) were available reporter genes (De Block et al., 1987; Jefferson et al., 1987; Jefferson, 1989; Spencer et al., 1990).

Different reporter genes are difficult to compare in general. A study by Töpfer et al. (1988) showed that the reporter genes varied among their sensitivity regarding the limit of detection. Here, GUS was shown to be the most sensitive reporter followed by NEO and CAT when analyzed in a transient protoplast system. In IME studies, it was shown that the reporter genes have an effect on the level of enhancement. The use of the CDS of *gusA* and, especially, the CDS of *cat* resulted in a stronger accumulation of RNA compared to the level of enhancement affected by the CDS of *luc* when IME of the *ZmAdh1-S* intron was studied in maize suspension cells (Luehrsen and Walbot, 1991). This effect was also observed when GUS and LUC activity was measured in transiently transformed green and etiolated rice seedlings, respectively (Morita et al., 2012). The *OsSodCc2* intron led to a 17-fold enhancement of GUS activity but only to a fivefold enhancement of LUC activity. Even though being less sensitive than GUS in transient expression systems (Töpfer et al., 1988), CAT was still used as a reporter genes for monocots in 2002 (Clancy and Hannah, 2002). In contrast, CAT was never used as a reporter gene studying IME in dicots. This is because CAT is an inefficient reporter in *Brassica* species as a matter of high endogenous CAT activity levels among this genus (Charest et al., 1989). Furthermore, both *B. napus* and *B. juncea* contain inhibitors of CAT that are predominantly acting on the bacterial CAT introduced into transgenic plants (Charest et al., 1989).

Phosphinothricin acetyltransferase confers resistance against herbicide and, thus, is primarily suitable for selecting transgenic plants that were transformed with *Agrobacterium* (Becker et al., 1992) and, therefore, only Rethmeier et al. (1997) used *pat* as a reporter gene alongside with *cat* to analyze the effect of the *OsSalT* intron in cell suspension cultures of maize.

Neomycin phosphotransferase II is still widely used as a selection marker conferring resistance to both neomycin and kanamycin (Brzezinska and Davies, 1973; Barton et al., 1983). The genomic sequence of *nptII* was mapped to a specific on the transposon T5 in bacteria in 1976 (Jorgensen et al., 1979). The reporter gene *nptII* was only used by Callis et al. (1987) to study the influence of different reporter genes on IME mediated by the maize *Adh1-S* intron (Callis et al., 1987).

By the time firefly LUC was described as an attractive reporter of gene expression (Ow et al., 1986) the advantage of this reporter over the herbicides CAT and PAT was that LUC enabled a fast screening of a large number of plants by luminescence. Additionally, LUC activity assay was reported to be over a 100-fold more sensitive than the CAT assay when gene expression was driven by the CaMV 35S promoter (Ow et al., 1986). The luminescence of LUC allowed a non-invasive detection of gene expression patterns that could even be assessed in the course of development of a plant. However, the substrate luciferin had to be taken up by the plants via the root system and caused expression patterns that were mainly determined by the track of uptake and, thus, the vasculature system (Ow et al., 1986). With the exception of the studies by Chung et al. (2006) and Morita et al. (2012) that also used sea pansy LUC (*Renilla reniformis*) as a reporter, all other studies used firefly LUC (*Photinus pyralis*) to monitor LUC activity in transient monocot (Luehrsen and Walbot, 1991; Washio and Morikawa, 2006; Morita et al., 2012) and dicot (Norris et al., 1993) systems and in stably transformed *Arabidopsis* plants (Chung et al., 2006) (**Figure 2**). However, the *Renilla luc* was used as an internal control in both publications. This was possible, because the main difference between both LUC enzymes is that they use different substrates, require different cofactors and emit light at different wave lengths (Wood, 1998).

The majority of experiments on IME was performed with *gusA* as a reporter gene. The advantage of using GUS as a reporter is that the GUS system is an attractive histochemical technique that enables the detection of enzyme activity directly in tissues (Jefferson et al., 1987). In contrast to LUC, the staining is evenly distributed through the tissue in *Brassicaceae* (Ow et al., 1986; Jefferson, 1989) and presumably the reason why the GUS system came out on top of the reporter genes used to study IME. The ability to monitor tissue-specific expression via GUS is tightly connected with the ability to easily transform dicots like tobacco and, especially, *Arabidopsis* via *Agrobacterium*-mediated transformation (Barton et al., 1983; Clough and Bent, 1998). This becomes apparent when comparing the long list of publications that report a function of IME in tissue-specific expression in dicots with the two examples listed for monocots (**Figure 2**).

In contrast to dicots in which most publications investigated IME in transgenic plants, IME in monocots was mainly analyzed in transient expression systems including protoplast, suspension cells, callus, and leaves (Vasil et al., 1989; Luehrsen and Walbot, 1991; Xu et al., 1994; Fiume et al., 2004). IME in transgenic monocots was only investigated in five publications (Xu et al., 1994; Jeon et al., 2000; Lu et al., 2008; Giani et al., 2009; He et al., 2009). In contrast to dicots, monocots are not the natural hosts of *Agrobacterium tumefaciens* (De Cleene and De Ley, 1976). The development of electroporation- and *Agrobacterium*-mediated transient gene expression systems provided a useful platform to study gene expression in plant cells (Fromm et al., 1985). This technique was the basis for evaluating IME in monocots mainly because regeneration of transgenic plants from transformed tissues was the major bottleneck for generating stably transformed plants for a long time (Sood et al., 2011). Differences in IME might also be related to the transgene copy number which is a matter of the transformation method used (Travella et al., 2005). However, a direct comparison of IME studied in transient and stable systems is as challenging as to compare IME in monocots and dicots. There is no example providing a direct comparison of an intron whose effect on gene expression was quantitatively tested side by side in a transient and a stable system. Nevertheless, rice *Rubi3* was investigated in two publications by the same group in transgenic rice plants, transgenic callus, and suspension cells derived from callus (Lu et al., 2008; Samadder et al., 2008). The studies showed that the effect of IME on gene expression is also dependent on the system used. The highest level of enhancement on mRNA and GUS activity level was observed for the actively dividing tissues (callus and suspension cells, respectively), while levels of enhancement were much lower in roots and leaves of transgenic rice plants. The levels of enhancement on mRNA level varied between 2.2-fold for leaves and 20-fold for suspension cells, those on GUS activity level between 3.3-fold for leaves and 51.1-fold for callus (Lu et al., 2008; Samadder et al., 2008).

## IME Affects Multiple Levels of Gene Expression

While the previous sections gave an overview on the complexity (**Figure 1**) and the methodology (**Figure 2**) this section will describe fundamental research of selected publications in more detail. Herein, the levels that are targeted by IME and its modes of action were ordered according to the different levels of gene expression, starting with transcription and ending with translation.

### Transcription

Because IME primarily affects mRNA accumulation and translation (Rose and Last, 1997), IME targeting active transcription was not investigated intensively. In general, the impact of IME on transcription does not exceed two- to threefold (Rethmeier et al., 1997; Rose and Last, 1997; Furger et al., 2002; Samadder et al., 2008; Moabbi et al., 2012; Laxa et al., 2016). Thus, a significant difference in transcription that is mediated by an intron compared to an intronless control is even harder to detect than an increase in mRNA accumulation by 20-fold or more. Among those publications that report an enhancement of transcription nearly all of them used the nuclear run-on (NRO) assay to detect nascent transcripts that are still attached to the actively elongating polymerase (Rethmeier et al., 1997; Rose and Last, 1997; Furger et al., 2002; Samadder et al., 2008; Moabbi et al., 2012). Furger et al. (2002) reported a twofold enhancement of transcription of a HIV-1 minigene in mammalian HeLa cells. In *Saccharomyces cerevisiae*, the *ACT1* intron was able to enhance transcription of the naturally intronless *INO1* gene by nearly threefold in the absence of the inducing agent inositol (Moabbi et al., 2012). In plants, an effect on transcription was first hypothesized for the rice *salT* intron in maize suspension cells (Rethmeier et al., 1997). The idea that the *salT* intron affects transcription was based on the observation that the intron elevated *cat* gene expression without affecting *cat* mRNA stability. Samadder et al. (2008) found a twofold increase in transcription of the rice *Rubi3* gene by IME in transgenic suspension cells. Besides the enhancement of transcription, the 5′UTR intron of *Rubi3* positively influenced mRNA levels by 20-fold and GUS activity by 29-fold, respectively. The same mode of action was observed for the *Arabidopsis PAT1* gene (Rose and Last, 1997). The presence of the two first introns of *PAT1* enhanced transcription of *gusA* below twofold but both mRNA accumulation and GUS activity by 30-fold in stably transformed *Arabidopsis* plants. The 5′UTR intron of glutamate:glyoxylate aminotransferase 1 (*GGT1*) enhanced transcription as indicated by a decrease in RNA polymerase II binding in the absence of the 5′UTR intron from a chimeric *gusA* construct (Laxa et al., 2016). In contrast to the above mentioned studies no further enhancement was seen on either mRNA or GUS activity level.

The fact that the expression of the *unc-54* gene in *C. elegans* entirely depends on the presence of its introns (Okkema et al., 1993), indicates that introns are able to initiate transcription, at least in nematodes. In plants, the question remains whether tissue-specific activation of an intron is a consequence of real transcriptional initiation, an enhancement of expression over background levels or a combination of both. In case of a real transcriptional initiation, *cis*-elements and an intrinsic promoter activity might be necessary enabling the recruitment of the transcriptional machinery to the promoter. In case of a simple enhancement, it is assumed that the expression in the specific tissue is already determined but too little to detect.

The fact that the 5′UTR intron of *GGT1* is able to drive leaf expression of both *GGT2* and *GLDP1-P2* as well as expression in trichomes of a root-specific *PEROXIDASE* (Laxa et al., 2016) might support the theory that expression is enhanced in a tissue in which expression is already determined but below the detection limit. Supporting this theory, Rose and Last (1997) showed that an increase in mRNA accumulation is depending on the presence of the *AtPAT1* intron, while there was no change in the rate of transcription when compared to the intronless construct (Rose and Last, 1997). On the other hand, the presence of the intron had a direct influence on RNA polymerase II binding to the chimeric *GGT1*::*gusA* construct indicating a recruitment of the transcriptional machinery to the promoter (Laxa et al., 2016).

Some introns contain an intrinsic promoter activity. This means that the intron is able to drive gene expression without a minimal promoter sequence present. In monocots, the maize *ubiquitin 1* intron can drive GUS expression in tritordeum inflorescences (Salgueiro et al., 2000). In dicots, an intron with reported intrinsic promoter activity is the intron of *Petunia* madbox gene *FBP11* (floral binding protein 11) which mediates GUS expression in floral organs including sepal, petal, stamen, carpel organs when fused to the *gusA* gene (Liao et al., 2013). The second example is the sesame *FAD2* gene that encodes a fatty acid desaturase. The intrinsic promoter activity of the 5′UTR intron drives very low levels of GUS expression in developing seeds of *Arabidopsis* (Kim et al., 2006). This intron can be seen as an example for the influence of an intron on tissue specificity. This is because it is able to overwrite the constitutive expression of the 35S promoter and specifically direct GUS expression to developing seeds (Kim et al., 2006). However, only quantitative GUS activity data are available that show *35S-SeFAD2-intron::gusA* expression is detectable in developing seeds. GUS stainings of plants carrying this construct were unfortunately not presented in this publication. A low intrinsic promoter activity was also shown for the 5′UTR intron of the *Brassica napus FAD2* gene in transgenic *Arabidopsis* (Xiao et al., 2014). Unfortunately, GUS staining is not homogeneous in the tissues analyzed in this publication. In addition, *35S-BnFAD2-intron::gusA* expression did not led to an exclusive expression of the 35S promoter in seeds but enhanced expression in all tissues analyzed (Xiao et al., 2014). This inconsistency can be explained by the fact that introns originating from different species can have different effects. In the end, this cannot be judged because GUS stainings of plants expressing *35S-SeFAD2-intron::gusA* are not available (Kim et al., 2006).

### Constitutive Expression Mediated by Introns

There are many examples in the literature that describe that constitutive expression of genes solely depends on the presence of a specific intron. The consensus among publications describing this phenomenon is that the presence of a specific intron often discriminates between expression in reproductive and vegetative tissues within a gene family. Constitutive expression of many genes encoding cytoskeleton proteins was shown to be regulated by introns. The gene family of *Arabidopsis* profilins contains five isoforms of which three (*PRF1*, *PRF2*, and *PRF3*) are expressed in vegetative tissues, while *PRF4* and *PRF5* are mainly expressed in pollen and, thus, are called reproductive profilins (Jeong et al., 2006). Jeong and colleagues found that the expression of *PRF1* and *PRF2*, representing the vegetative profilins, is solely mediated by the first intron. The *PRF2* promoter itself drove vascular expression, while the addition of the first intron to the constructs strongly activated GUS expression in the whole plant. When the first intron of the reproductive *PRF5* was replaced by the *PRF2* intron, strong GUS expression was observed throughout plants. Conversely, a swap construct, in which the endogenous first intron of *PRF2* was replaced by the *PRF5* intron, mimicked the GUS expression pattern of *PRF2* lacking its first intron. This indicated that vegetative expression of profilins was not only dependent on the presence of the intron alone, but must also be attributed to specific sequences within the intron of *PRF2* that are missing in *PRF5* (Jeong et al., 2006). Activation of *PRF5* in vegetative tissue is not restricted to introns originating from the same gene family. Vegetative expression of *PRF5* can also be mediated by the first intron of *Petunia ADF1* (actin depolymerizing factor 1) in stably transformed *Arabidopsis* plants (Jeong et al., 2007). In addition, this experiment strengthened the hypothesis that IME is evolutionary conserved among different plant species. As observed for *PRF2*, the *PhADF1* promoter conferred expression in the vascular system, while the intron activated expression in vegetative tissue in *Arabidopsis* (Mun et al., 2002). The *Arabidopsis* genes *ACT1* and *ACT2* represent vegetative and reproductive actin genes, respectively (Meagher et al., 1999). Intron deletion analysis showed that the expression of *ACT1* in pollen is strongly enhanced by the presence of its first intron. However, substituting the *ACT1* intron by the first intron of *ACT2* led to a strict repression of GUS activity in pollen (Vitale et al., 2003).

Besides introns that mediate constitutive expression of genes encoding cytoskeleton proteins, there is a variety of other examples. The expression of the replication-dependent histone H4 is meristem-specific and restricted to the S-phase of the cell cycle. Cloning the first intron of the replacement histone H3 downstream of the H4 promoter led to a constitutive expression in *Arabidopsis* plants (Chaubet-Gigot et al., 2001). Thus, the intron is able to overwrite the tissue specificity that is determined by the promoter. The *Arabidopsis COX5c-2* gene encodes the subunit 5c of the mitochondrial cytochrome c oxidase and contains a 5′UTR intron. As a component of the mitochondrial electron transport chain one does assume a high expression of *COX5c-2* in meristems and actively growing tissues. An intron deletion experiment confirmed that this expression pattern can be attributed to the 5′UTR intron of *COX5c-2*. The promoter alone only drove *gusA* expression in pollen (Curi et al., 2005). When fused between the *COX5b-1* promoter and the *gusA* gene, the 5′UTR intron of *COX5c-2* was also able to drive leaf expression of *COX5b-1* which is naturally expressed in the vascular system. A similar observation was made for *AtPUX7*. While the promoter itself drove GUS expression in the early male gametophyte, the addition of the first intron-mediated strong GUS expression in whole seedlings which the authors called sporophytic expression (Gallois et al., 2013).

### IME Determines Tissue Specificity and Spatial Expression of Genes

In *Arabidopsis*, the 5′UTR intron of the *SUVH3* gene, a *Su(var)3-9* homolog encoding a SET domain protein with H3K9 methyltransferase activity, was shown to be required not only for maximum GUS expression but also to confer tissue-specific expression in roots, leaves, and flowers (Casas-Mollano et al., 2006). The tissue-specific expression of the *Petunia* mad box gene *FBP11* is regulated by both the promoter and the first intron (Liao et al., 2013). The promoter alone drove expression in vegetative and floral tissue, while the intron possessed an intrinsic promoter activity and mediated expression in floral organs including sepal, petal, stamen, and carpel organs. However, the combination of promoter and intron only drove GUS expression in ovary and carpel tissue. Giani et al. (2009) reported an intron-dependent spatial expression for rice *OsTub4*. In this study, the intron-mediated a specific GUS expression pattern in nodes, internodes, and leaves. For example, specific GUS expression changed from central vessels to the outer parenchyma in rice plants when the intron was absent. A similar observation was made for rice *Tua1* whose intron directs gene expression to actively dividing tissues like root tips (Jeon et al., 2000).

Introns were shown to mediate expression in all tissues of the roots. In *Arabidopsis*, the *UBQ10* intron conferred GUS expression of *CNGC2*, *YAB3*, *GAE1*, *ROP10*, *ADL1A*, *MSBP1*, and *ULI3* in roots (Emami et al., 2013). Karthikeyan et al. (2009) reported that the 5′UTR intron of *Arabidopsis PHT1;4* (encoding a high affinity phosphate transporter) is essential for *PHT1;4* expression in root tips and for an increase in expression during phosphate starvation (Karthikeyan et al., 2009). This regulation is quite specific in terms of that the response to phosphate limitation is mediated by an intron and, thus, translates an environmental signal into gene expression.

Screening the literature on IME targeting tissue specificity and spatial expression of genes it is striking that the majority of publications show only one representative GUS staining image of plants and/or tissues that have been analyzed. In many publications not even the number of individual transformation events is evident. However, the variation between either transformation events or different experiments can be quite high as seen for transgenic lines (Snowden et al., 1996; Chung et al., 2006; Laxa et al., 2016). Sometimes results are difficult to judge when only specific tissues are affected by the presence of an intron (Giani et al., 2009; Karthikeyan et al., 2009).

### Post-Transcription and Translation

A few publications distinguished between effects on mRNA accumulation and translation. Even though the *rice Ubi3* 5′UTR intron already led to an increase in mRNA accumulation by 20-fold, its enhancing effect was even higher when protein activity (29-fold enhancement) was measured in rice suspension cultures (Samadder et al., 2008). Thus, the intron must also affect the translational level. The same effect was observed in transgenic rice plants transformed with the *rice Ubi3* intron (Lu et al., 2008). Additionally, this publication demonstrated that the effect of IME on translation is tissue-dependent. While *gusA* mRNA accumulated to 2.2-, 12.8-, and 17-fold in leaf, root, and callus tissue, protein activity was enhanced by 3.3-, 26.5-, and 51.1-fold, respectively. Thus, it can be assumed that translation is differently regulated in different tissue (Kawaguchi and Bailey-Serres, 2002). Mascarenhas et al. (1990) observed an increase in *cat* mRNA by 3.9-fold and in CAT activity by 12.1-fold indicating that IME mediated by the maize *Adh1* intron 2 is also targeting two different regulatory levels in maize protoplasts.

## Mechanisms of IME

Intron-mediated enhancement is determined by a variety of parameters (**Figure 1**). As described in the introduction the main requirements of IME is that the intron has to be located within the transcribed sequence and close to TIS in the correct orientation in monocots (Callis et al., 1987; Vasil et al., 1989; McElroy et al., 1990; Maas et al., 1991; Clancy et al., 1994; Snowden et al., 1996) and dicots (Gidekel et al., 1996; Chaubet-Gigot et al., 2001; Mun et al., 2002; Rose, 2002, 2004; Jeong et al., 2006, 2007; Akua et al., 2010). Furthermore, IME strongly depends on splicing in monocots (Mascarenhas et al., 1990; Clancy et al., 1994; Clancy and Hannah, 2002; Morello et al., 2011) but not in dicots (Rose and Beliakoff, 2000; Jeong et al., 2007), with the exception of the *AtMHX* intron (Akua et al., 2010). Additionally, IME is affected by the length and composition of flanking sequences, the promoter and CDS (Callis et al., 1987; Luehrsen and Walbot, 1991; Rethmeier et al., 1997; Jeon et al., 2000; Chaubet-Gigot et al., 2001; Mun et al., 2002; Vitale et al., 2003; Akua et al., 2010). **Table 1** summarizes the IME requirement and mechanism according to their discovery. Even though the mechanism of IME is still not fully resolved, the main requirements of how the intron has to be positioned to enhance transcription, that splicing is a prerequisite in monocots and that flanking sequences, promoter sequences, and the CDS affect the level of enhancement by a special intron was uncovered within the first 5 years after the discovery of IME in plants (**Table 1**). Interestingly, these findings based on IME studies in monocots. With the exception of the necessity of splicing, the basic requirements of IME were also described in dicots. Taken into consideration that IME in monocots was primarily investigated in transient transformation systems, while experiments on IME in dicots based on stably transformed plants, one can assume that these requirements generally apply for introns involved in IME. In the late 1990s, the focus to unravel the mechanism changed from monocots to dicots. This can be related to the fact that in dicots IME is not entirely depending on splicing as observed in monocots. Furthermore, sequence elements within dicot introns might play a primary role for IME. Regions important for IME were analyzed by deletion studies in several publications (Rose, 2002; Chung et al., 2006; Kim et al., 2006; Akua and Shaul, 2013; Xiao et al., 2014). But the majority of publications describes the phenomenon of IME by a special intron and, in some cases, assigns IME either to a special region within the intron or to the splicing event of the intron. However, evidence for unraveling the mechanism of IME on the different levels is rare.

**Table 1:** Chronological summary of the discovery of the requirements and mechanisms of intron-mediated enhancement (IME) in plants.

Years	Intron	Species	IME requirement/mechanism	References
1987	*ZmAdh1*	Monocot	First report IME in plants, located in CDS, close to TIS, promoter and CDS makes a difference	Callis et al., 1987
1989	*ZmSh1*	Monocot	Sense orientation, enhancement dependent on plant species	Vasil et al., 1989
1990	*OsAct1, ZmAdh1*	Monocot	Splicing required	McElroy et al., 1990; Mascarenhas et al., 1990
1990	*ZmAdh1*	Monocot	Effect on mRNA accumulation as well as translation	Mascarenhas et al., 1990
1990	*RcCAT1*	Dicot	IME by a dicot intron in monocot	Tanaka et al., 1990
1991	*ZmAdh1*	Monocot	Length and composition of flanking sequence are important	Luehrsen and Walbot, 1991
1991	*ZmSh1*	Monocot	Proposed post-transcriptional mechanism via splicing, monocot intron does not work in dicot	Maas et al., 1991
1994	*ZmSh1*	Monocot	IME acts on multiple levels of gene expression, large intron deletions can be made without significantly altering activity	Clancy et al., 1994
1997	*AtTWN2*	Dicot	IME affects gene expression diversely in different tissues	Zhang and Somerville, 1997
1997	*AtPAT1*	Dicot	Proposed post-transcriptional mechanism (mRNA only accumulating when intron present)	Rose and Last, 1997
2000	*AtPAT1*	Dicot	Splicing not required	Rose and Beliakoff, 2000
2002	*ZmSh1*	Monocot	35 bp, T-rich region important for enhancement and splicing	Clancy and Hannah, 2002
2002	*AtPAT1*	Dicot	T- (U-) rich intron regions are more important for IME than the overall T- (U-) content of the intron	Rose, 2002
2008		Monocot/Dicot	IMEter, bioinformatics analysis of sequence motifs supporting IME	Rose et al., 2008
2016	*AtGGT1*	Dicot	IME affects RNA polymerase II binding	Laxa et al., 2016

*The table gives information on the year of discovery, the intron that was analyzed, the species (monocot or dicot), and details on both the requirements and mechanisms that were reported. CDS, coding sequence.*

In mammals, Furger et al. (2002) proposed that splicing signals in the proximity to the promoter can directly influence gene transcription. This hypothesis is supported by the interaction between the U1 snRNA and the transcription factor TFIIH which enables a re-initiation of transcription in HeLa cells (Kwek et al., 2002). TFIIH is part of the pre-initiation complex and executes multiple functions including DNA-dependent ATPase, ATP-dependent DNA-helicase, and serine/threonine kinase activity (reviewed in Nikolov and Burley, 1997). TFIIH phosphorylates the C-terminus of RNA polymerase II and, thus, plays an important role in enabling active processing of the polymerase (Lu et al., 1992). Recently, Gallegos and Rose (2015) proposed a model in which the intron enhances transcript (re-) initiation within a distinct upstream region. In this model, transcript initiation is linked to specific sequences within introns instead of splicing factors. This model is consistent with the necessity of introns being located in the proximity to the transcription initiation site (TIS). Furthermore, this model does not interfere with the requirement of intron splicing which has been observed in monocots and might even provide another level of regulation (Gallegos and Rose, 2015).

A model in which specific sequences within introns are involved in transcript initiation is supported by a few publications in which both *cis*-elements within introns and intron interacting factors were described. In *Arabidopsis*, the spatial and temporal expression of *AGAMOUS* (*AG*) which is involved in the development of flowers was shown to be mediated by an intragenic region (Sieburth and Meyerowitz, 1997). The intragenic region was localized to a 91 bp region in the second intron of *AG* that was able to bind the transcription factor LEAFY (LFY; Busch et al., 1999). Lohmann et al. (2001) found that LFY interacts with the homeodomain protein WUSCHEL (WUS). Together, they activate *AG* expression in the center of flowers. The binding sites for LFY [CCAATG(G/T)] and WUS [TTAAT(G/C)(G/C)] were both found twice in the *AG* intron sequence in an orientation in which the LFY-binding sites are flanked by the WUS-binding sites (Lohmann et al., 2001). In monocots, the maize *ubiquitin 1* intron can drive GUS expression in tritordeum inflorescences (Salgueiro et al., 2000). The authors highlighted an *Opaque-2* binding motif as being part of the functional intron. However, there is no experimental evidence that this motif is of direct functional relevance within the *ubiquitin 1* intron. Opaque-2 was shown to be involved in the regulation of albumin b-32 and zein proteins in the endosperm (Soave et al., 1981; Lohmer et al., 1991). In *opaque-2* mutants, zein proteins were shown to only accumulate to 50–70% of the wild type level. Further studies proved that the reduction in transcript levels can be directly correlated to transcriptional activation as shown by nuclear run on assays (Kodrzycki et al., 1989). Thus, the *Opaque-2* binding motif in the *ubiquitin 1* intron might play a possible role in the *gusA* transcript initiation in tritordeum inflorescences described by Salgueiro et al. (2000). Introns were classified according to their IMEter scores. This bioinformatic approach is based on the observation that the nucleotide composition of introns located close to TIS is different to those located further downstream in the gene body. The analysis revealed two similar motifs in *Arabidopsis* with the consensus sequences TTNGATYTG (Rose et al., 2008) and CGATT (Parra et al., 2011). A very recent publication investigated the transformation of an intron with a small IME effect into one having a strong impact on mRNA accumulation (Rose et al., 2016). Both motifs were shown to increase mRNA accumulation with TTNGATYTG being more active. The combination of both led to a reduction of enhancement. However, no interacting proteins that bind to these motifs were identified yet. Recently, the non-coding RNA *HIDDEN TREASURE 1* (*HD1*) was shown to interact with the 5′UTR intron of the PHYTOCHROME INTERACTING FACTOR 3 (*PIF3*; Wang et al., 2014). The interaction is essential for the downregulation of *PIF3* in response to red light and, thus, for the control of photomorphogenesis.

In *S. cerevisiae*, introns were shown to act on transcript initiation by a mechanism called gene looping (El Kaderi et al., 2009). The model of gene looping postulates a close proximity between the promoter and the terminator of a gene. The advantage of this clustering is that by the time the RNA polymerase II reaches the terminator region, transcription can be immediately re-initiated (El Kaderi et al., 2009). However, gene looping would also bring together transcription factors and RNA polymerase II. Thus, this mechanism might not only dependent on an interaction between the promoter and terminator as suggested by the authors. Taking into consideration that enhancing introns are located close to the 5′ end of a gene (Rose et al., 2008) one could assume that the mechanism of gene looping would help to increase transcript initiation rather than re-initiation. Besides RNA polymerase II recycling, intron-mediated promoter directionality was shown to be closely related to gene looping. Based on the knowledge that the promoters of many RNA polymerase II transcribed genes function bidirectional, Agarwal and Ansari (2016) tested whether the presence of an intron has an impact on the abundance of non-coding RNAs (uaRNA, upstream antisense RNA) and coding RNAs (mRNA). They found that the presence of an intron favors the transcription of mRNA over uaRNA. The inhibition of uaRNA in the presence of an intron has been demonstrated to be a consequence of the recruitment of termination factors in close vicinity of the promoter.

An additional aspect of how introns might influence transcription is based on the observation that active histone modifications like H3K9 acetylation and H3K4me3 trimethylation are enriched at first exon-intron borders in human genes (Bieberstein et al., 2012). Having a closer look on activating histone modifications and the IMEter score in *Arabidopsis*, Gallegos and Rose (2015) found a similarity of their distributions relative to TIS. Based on this, the authors hypothesized that introns could influence transcript initiation by acting on the chromatin structure. In terms of chromatin structure, this does not only involve activating histone modifications but also the nucleosome density which is preferentially low in the proximity to TIS (Ha et al., 2011; Gallegos and Rose, 2015). Gallegos and Rose (2015) suggested that an increase in RNA polymerase II processivity could be an explanation for the positive influence of introns on mRNA accumulation (Gallegos and Rose, 2015). This model indicates that splicing signals are important for IME function and is supported by the finding that a direct interaction was shown for U1 snRNA and the transcription factor TFIIH in HeLa cells (Kwek et al., 2002). Supporting this model, the 5′UTR intron of *Arabidopsis GGT1* was shown to regulate maximum transcript abundance by recruiting RNA polymerase II (Laxa et al., 2016). However, a direct interaction between splicing factors and RNA polymerase II was not tested. Furthermore, splicing was shown to be a co-transcriptional process (Custódio and Carmo-Fonseca, 2016) in which phosphorylated, actively describing RNA polymerase II influences pre-mRNA splicing (Hirose et al., 1999). Simultaneously, an impact of introns stimulating RNA polymerase processivity and, thus, transcript elongation can be hypothesized.

In some cases, the mechanism of IME is tightly linked to the splicing event of the enhancing intron. This phenomenon was mainly reported in monocots (Luehrsen and Walbot, 1991; Sinibaldi and Mettler, 1992; Akua et al., 2010) but is not required for IME in general (Rose and Beliakoff, 2000). As described above, the presence of an intron is accompanied with the recruitment of splicing factors. Splicing was shown to positively influence polyadenylation, capping, transcript stability, and translation (Proudfoot et al., 2002). Wiegand et al. (2003) provided evidence that the exon junction complex (EJC) is primarily responsible for IME in human cell lines. Furthermore, it has been suggested that the EJC increases the efficiency of nuclear export of transcripts and their translation (Le Hir et al., 2001).

## Foreign Gene Expression Can Mainly Benefit from IME Targeting mRNA Stability and Translation

Because introns are known to enhance gene expression on the level of transcription, post-transcription, and translation (Samadder et al., 2008), it can be questioned whether the use of introns might help to increase gene expression in transgenic plants. Some introns are already in use for biotechnological approaches, especially in monocots. This is due to the fact that the Cauliflower mosaic virus (CaMV) 35S promoter is less effective in monocots (Mitsuhara et al., 1996). In maize, introns of endogenous maize genes like *Adh1*, *Sh1*, *Ubi1*, and *Act1* were tested side by side with an intron from the chalcone synthase gene from *Petunia* in combination with the CaMV 35S promoter (Vain et al., 1996). The highest level of enhancement was reported for the maize *Ubi1* intron. This 5′UTR intron in combination with its respective promoter is most commonly used in biotechnology (Scott, 2009) and was already used successfully to express the two diagnostic proteins avidin and β-glucuronidase, respectively (Hood et al., 1997; Witcher et al., 1998). An increase in efficiency in gene expression was also shown for transgenic rice when the maize *Ubi1* promoter and intron were used (Christensen and Quail, 1996). A general feature of these promoters is their constitutive expression in plant tissue.

A downside of biotechnological approaches is that foreign gene expression that is driven by either strong constitutive promoters or multiple copies of the transgene in the plant genome are prone to gene silencing via co-suppression (Depicker and Van Montagu, 1997). Weaker promoters and tissue-specific expression might overcome this problem of silencing. Because IME affects different levels such as mRNA stability (Rose and Beliakoff, 2000) introns that mainly target later stages in transcription can be an excellent tool to enhance expression of genes in combination with weak promoters. Additionally, the effect of enhancement is even stronger when a weak promoter is used (Callis et al., 1987). The low level of enhancement of IME on active transcription does not qualify introns for a direct application in foreign gene expression. However, because IME of a specific intron often targets different levels (Rose and Last, 1997; Samadder et al., 2008) an intron with a low level of enhancement on transcription can be of interest because it might stabilize mRNA, translation or both.

Additionally, a high constitutive expression of foreign genes is often not that what is beneficial for the expression of foreign genes in plant expression systems (Scott, 2009; Desai et al., 2010). The most important arguments for a tissue-specific expression are (i) transgene expression should not be active in pollen, (ii) insecticidal proteins should not be expressed in grains, and (iii) specific gene expression in seeds and tubers simplifies storage and isolation of the proteins. A recent review by Dutt et al. (2014) summarizes detailed information on promoters that determine temporal and spatial gene expression in various crop species (Dutt et al., 2014). For example, maize endosperm-specific promoters that are commonly used are maize 27 kDa zein (*zmZ27*), maize *waxy* (starch synthase) genes, rice *glutelin-1*, and rice ADP-glucose pyrophosphorylase small subunit gene (Zheng et al., 1993; Russell and Fromm, 1997). Even though these genes are mainly endosperm-specific, low level activity in other tissue might still be detected as observed for the maize *waxy* promoter in pollen (Russell and Fromm, 1997).

Introns function by having a positive influence either on mRNA maturation or on transcript stability (Rose and Beliakoff, 2000). Some publications reported an increase in steady-state levels of mature mRNA (Callis et al., 1987; Rethmeier et al., 1997; Rose and Last, 1997) that can be independent of an increase in mRNA stability (Rethmeier et al., 1997). In the presence of an intron mRNA accumulates up to 100-fold and more compared to the intronless control. The highest levels of enhancement were shown for introns from monocots like the maize *Sh1* (*shrunken-1*) gene that enhances CAT activity in rice and maize protoplast by 100-fold (Maas et al., 1991). In combination with the first exon of *Sh1* the intron increases CAT activity even by 1000-fold. However, when this intron was tested in tobacco protoplast no enhancement was observed (Maas et al., 1991). Thus, the monocot intron fails to increase expression in a dicot. This is due to the fact that the GC content of monocot introns is higher than that of dicot introns (White et al., 1992; Singh et al., 2016). Furthermore, splicing of dicot introns necessitates AU-rich sequences (Goodall and Filipowicz, 1991). Therefore, the selection of an appropriate intron for the design of expression vectors for plant transformation also depends on the destination plant. Thus, foreign gene expression in dicots might be limited by the low number of introns with high enhancing activities that have been characterized to date. However, the example of *Arabidopsis ATPK1* showed that a high level of enhancement is also possible in transgenic dicots (Zhang et al., 1994).

Tissue-specific and spatial expression of introns is a combination of introns and their respective intron sequences. Thus, a direct and, in particular, a predictable application of intron sequences in established expression systems will be difficult. Detailed and costly analysis of how a specific intron influences gene expression of target genes in transgenic plants would be inevitable. Especially tissue-specific expression in roots would improve established expression systems. Since vascular plants evolved, roots are important organs that anchor the plant, enable nutrient and water supply as well as storage (Schiefelbein and Benfey, 1991; Raven and Edwards, 2001). Additionally, roots are the organs at which plants establish symbiosis with mycorrhizal fungi (Hollaender et al., 1977). Introns that mediate root-specific expression could help to enhance gene expression of established root-specific promoters and, thereby, improve the transport of nutrients to the storage organs like tubers. Introns like *ACT2* that led to a strict repression of GUS activity in pollen (Vitale et al., 2003) could be of great biotechnological interest to optimize risks of contamination of transgene expression in pollen.

A drawback for the application of introns to be used for foreign gene expression in plants is that the mechanism of IME is far from being resolved. As mentioned before, a direct and, in particular, predictable application of intron sequences in established expression systems would be difficult and costly. Once the mechanism of IME and responsible sequence elements will be identified one can easily edit introns of target genes by genome editing technologies like CRISPR/Cas and TALENs, respectively (Beurdeley et al., 2013; Chen and Gao, 2013; Khatodia et al., 2016; Zhang et al., 2016). These techniques enable positional editing of the plant genome and can be used to introduce or delete IME motifs in specific introns thereby changing mRNA accumulation, tissue specificity or spatial expression of endogenous genes. In general, engineering introns for plant biotechnology necessitates a more detailed characterization of the underlying mechanism of IME of the most promising introns. Especially chimeric constructs need special attention in characterization, simply because in many cases tissue-specific and spatial expression is determined by both promoter and intron sequences.

## Conclusion

The use of IME for foreign gene expression in plants has many advantages but also limitations. Because IME mainly targets both mRNA accumulation and translation introns are great tools to optimize gene expression. To date, foreign gene expression in plants is limited by low levels of protein accumulation. IME is a potential tool to overcome this limitation. At the same time it can minimize the risk of gene silencing because relatively weak promoters can be used. Some introns even have the potential to further optimize tissue-specific expression of established gene expression systems by repressing transgene expression for example in pollen and, thus, preventing contaminations. For a direct application, it needs to be taken into consideration that monocot introns are not efficiently spliced in dicots. Additionally, IME is also dependent on the length and composition of adjacent sequences. Detailed information of how IME works would be advantageous to predict intron function in different plant systems and help to select the best possible intron for a specific transgene. The different levels and proposed mechanisms of IME action are still poorly understood. However, the variety of IME targeting different levels and being regulated by different mechanisms at the same time is not only a downside for gene expression but also an enormous tool box for a flexible optimization of gene expression.

## Author Contributions

The author confirms being the sole contributor of this work and approved it for publication.

## Conflict of Interest Statement

The authors declare that the research was conducted in the absence of any commercial or financial relationships that could be construed as a potential conflict of interest.
